# Therapeutic potential of ADSCs in diabetic wounds: a proteomics-based approach

**DOI:** 10.3389/fcell.2024.1468220

**Published:** 2024-09-13

**Authors:** Yuan Gu, Zelan Mu, Yuanzheng Chen, Can Wu, Jie Shi, Nan Bai

**Affiliations:** ^1^ School of Clinical Medicine, Shandong Second Medical University, Weifang, China; ^2^ Department of Burns and Plastic Surgery, Emergency General Hospital, Beijing, China; ^3^ Medical Cosmetic Plastic Surgery, Linyi People′s Hospital, Linyi, China; ^4^ Plastic and Cosmetic Surgery, People′s Hospital of Liaoning Province, Shenyang, China

**Keywords:** diabetic wounds, proteomics, ADSCs, bioinformatics, chronic wounds

## Abstract

**Background:**

Diabetes mellitus (DM), a chronic metabolic disease characterized by elevated blood sugar, leads to delayed or non-healing wounds, increasing amputation risks, and placing a significant burden on patients and society. While extensive research has been conducted on adipose-derived stem cells (ADSCs) for promoting wound healing, there is a scarcity of studies focusing on diabetic wounds, particularly those employing proteomics and bioinformatics approaches.

**Objective:**

This study aimed to investigate the mechanisms by which ADSCs promote diabetic wound healing using proteomics and bioinformatics techniques.

**Methods:**

Healthy rat fat tissue was used to isolate ADSCs. A T2DM rat model with back wounds was established. The experimental group received ADSC injections around the wound, while the control group received PBS injections. Wound healing rates were documented and photographed on days 0, 3, 7, 10, and 14. On day 7, wound tissues were excised for HE and Masson’s staining. Additionally, on day 7, tissues were analyzed for protein quantification using 4D-DIA, with subsequent GO and KEGG analyses for differentially expressed proteins (DEPs) and protein-protein interaction (PPI) network analysis using STRING database (String v11.5). Finally, Western blot experiments were performed on day 7 wounds to verify target proteins.

**Results and Conclusions:**

In all measured days postoperatively, the wound healing rate was significantly higher in the ADSC group than in the PBS group (day 7: *p* < 0.001, day 10: *p* = 0.001, day 14: *p* < 0.01), except on day 3 (*p* > 0.05). Proteomic analysis identified 474 differentially expressed proteins, with 224 key proteins after PPI analysis (78 upregulated and 146 downregulated in the ADSC group). The main cellular locations of these proteins were “cellular anatomical entity” and “protein-containing complex”, while the biological processes were “cellular processes” and “biological regulation”. The primary molecular functions were “binding” and “catalytic activity”, with GO enrichment focused on “Wnt-protein binding”, “neural development”, and “collagen-containing extracellular matrix”. Further analysis of PPI network nodes using LASSO regression identified Thy1 and Wls proteins, significantly upregulated in the ADSC group, as potentially crucial targets for ADSC application in diabetic wound treatment.

## 1 Introduction

Diabetes mellitus has emerged as the third most prevalent non-communicable disease globally, following cardiovascular and cerebrovascular diseases and cancers. Notably, over 90% of these cases are classified as type 2 diabetes mellitus (Nitzan et al., 2015). Diabetic foot and diabetic wounds are well-recognized complications associated with DM. Delayed and non-healing wounds can lead to ulceration, potentially progressing to amputation and even death in severe cases (Liu et al., 2021). These complications pose a significant threat to patient wellbeing and survival while also placing a substantial burden on both patients’ families and society as a whole (Cho et al., 2019). Current primary clinical treatment strategies for diabetic wounds encompass surgical debridement, meticulous wound care, anti-inflammatory therapy, and interventions to enhance blood supply and create an environment conducive to tissue regeneration (Everett and Mathioudakis, 2018). Despite these efforts, the majority of treatment options have not achieved optimal clinical outcomes, evidenced by persistently high rates of amputation for diabetic foot ulcers (DFU).

In recent years, researchers have investigated adult stem cells, particularly mesenchymal stem cells (MSCs), in the clinical treatment of type 2 diabetes mellitus due to their properties of easy accessibility, multidirectional differentiation, and self-renewal. This approach has yielded preliminary evidence of therapeutic benefit, raising hope for potential future treatment options for type 2 diabetes mellitus (Kong et al., 2014; Lei et al., 2014). Adipose-derived stem cells (ADSCs) possess self-renewal ability and multidirectional differentiation potential. Due to the easy accessibility and wide availability of adipose tissue, ADSCs are considered a more favorable option compared to bone marrow mesenchymal stem cells (BMSCs). ADSCs can influence fibroblasts, macrophages, endothelial cells, and keratinocytes, regulating wound inflammation, enhancing their proliferation and migration, inhibiting apoptosis, promoting neovascularization, and accelerating wound healing. Additionally, they stimulate re-epithelialization of the wound skin and improve scar formation (Ma et al., 2019a; Xu et al., 2019). Proteomics is a relatively new field that strives to comprehensively understand the dynamics of intracellular protein composition, expression levels, and modification states. It is a valuable tool for identifying protein interactions and associations, as well as for elucidating protein characteristics and cellular physiological processes (Savage, 2015). Consequently, proteomic research can provide a theoretical framework for investigating the pathogenesis of diseases like diabetes and the mechanisms by which ADSC treatment promotes the healing of diabetic wounds (Wang et al., 2018).

This study employed 4D-DIA quantitative proteomics to screen and identify differentially expressed proteins within the granulation tissue of diabetic rat back wounds following ADSC treatment. Bioinformatics analyses were then conducted to elucidate key proteins present in the granulation tissue and signaling pathways critical for wound healing. The findings from this investigation hold promise for the identification of specific biomarkers associated with ADSC-mediated diabetic wound healing.

## 2 Materials and Methods

### 2.1 Animal origin

Four to six-week-old male Sprague-Dawley (SD) rats (SPF grade), weighing 90–120 g, were used in this study. All ethical considerations and protocols for animal experiments were in accordance with the regulations of the Animal Management Committee of Linyi People’s Hospital.

### 2.2 Major reagents and equipments

The following reagents were utilized in the study: 0.15% collagenase I (Solepol), DMEM high glucose medium (Solepol), 10% fetal bovine serum (Solepol), penicillin-streptomycin double antibody (Zhongqiao Xinzhou), 0.25% trypsin (Gibco, United States), APC anti-rat CD45 antibody (BioLegend), PE anti-rat CD90.1 (Thy-1.1) antibody (BioLegend), FITC anti-rat CD29 antibody (BioLegend), lipogenic medium (Bodho Bio), osteogenic medium (Bodho Bio), Red oil O staining solution (Solepol), Osteoblast mineralized nodule staining kit (Alizarin Red S method) (Biyun Tian), streptozotocin (STZ) (Soluble), sodium citrate buffer (0.1 mol/L, pH 4.5) (Soluble), rabbit multi-anti GPR177 (62KD) (Wuhan Three Eagles Biotechnology Co., Ltd.), and rabbit multi-anti THY1 (28KD) (Affinity Corp.).

The following equipment was employed in the study: a CO2 constant-temperature incubator (Thermo Fisher Scientific LLC), an orthoptic microscope (Nikon Fi3 Biological, China), an inverted microscope (Nikon), a low-speed centrifuge (Hunan Kecheng Instrument Co., Ltd.), a flow cytometer (Beckman-Coulter), a Vortex-Genie two vortex mixer (SI, United States), a Centrifuge 5424 R high-speed refrigerated centrifuge (Eppendorf, Germany), an ABS-MS-078 thermostatic mixer (Hefei Ebenson Scientific Instrument Co., Ltd.), a Spectra Max plus384 enzyme marker (Molecular Devices, United States), an LNG-T98 frozen centrifugal concentrator and dryer (Taicang Huamei Biochemical Instrument Factory), an LNG-T88 Benchtop Rapid Centrifugal Concentration and Drying instrument (Taicang Huamei Biochemical Instrument Factory), a Tanon-3500R Automatic Digital Gel Image Analysis System (Shanghai Tannen Technology Co., Ltd.), an EPS-300 Digital Pressure and Current Stabilized Electrophoresis apparatus (Shanghai Tannen Technology Co., Ltd.), an EASY-nLC 1200 High Performance Liquid Chromatography system (Fisher), a timsTOF Pro tandem mass spectrometer (Bruker, Germany), an MS105DU analytical balance (Mettler Toledo, United States), and a Fielda-650D ultrasonic cell crusher (Jiangsu Wavefield Intelligent Science and Technology Co., Ltd.).

### 2.3 Isolation, culture, and characterization of ADSCs

Eight to ten-week-old male Sprague-Dawley (SD) rats (SPF grade), weighing 200–240 g, were used in this study. Following anesthesia, epididymal fat pads were excised. The adipose tissue was thoroughly washed with sterile phosphate-buffered saline (PBS) under aseptic conditions to remove any residual blood vessels and connective tissues. The tissue was then meticulously dissected with sterile scissors into small pieces. The resulting tissue fragments were digested with 1% type I collagenase solution (at a 1:1 ratio with the tissue volume) for 45 min at 37°C with continuous agitation (1000 rpm). Subsequently, the suspension was centrifuged at room temperature for 5 min. The cell pellet was then resuspended in fresh medium and centrifuged again under the same conditions. The isolated cells were uniformly seeded onto 6-well plates and cultured at 37°C in a humidified incubator with 5% CO_2_. After a 48-h incubation period, adherent cells were observed. Thereafter, the culture medium was replaced every 2–3 days. Once cell confluence reached approximately 80%–90%, cells were passaged at a 1:3 ratio. The third passage of ADSCs was used for subsequent experiments.

ADSCs were first observed for morphology under an inverted microscope. To assess their multipotency, third-generation ADSCs were cultured in plates with coverslips containing complete DMEM medium at 37°C and 5% CO2 until adherent. The medium was then replaced with either adipogenic or osteogenic induction medium to induce differentiation into adipose or bone cells, respectively. After 3 weeks, the cells were stained with oil red O and alizarin red to evaluate their differentiation potential, followed by microscopic analysis. Flow cytometry was employed to quantify the expression of specific surface markers (CD105, CD90, and CD29) on third-generation ADSCs. Briefly, cells were digested with 0.25% trypsin (without EDTA), collected by centrifugation at 1500 rpm for 5 min, and washed twice with PBS. The cell pellet was then resuspended in 100 µL of PBS, transferred to flow cytometry tubes, and incubated with a cocktail of antibodies (2 µL CD29-FITC, 0.5 µL CD90-PE, 5 µL CD45-APC and 1.25 µL of CD90-PE antibody) for 30 min at 4°C in the dark. Following another centrifugation and supernatant removal, the cells were washed again with PBS, resuspended in 200 µL PBS and analyzed by flow cytometry.

### 2.4 Establishment of SD diabetic rat back wound model and wound treatment

① Preparation of STZ Working Solution: The appropriate amount of STZ powder was weighed on an electronic balance. It was then placed in an EP tube wrapped in aluminum foil and stored in a dry, low-temperature environment. Before injection, STZ powder was dissolved in pre-cooled sodium citrate buffer (0.1 mol/L, pH 4.3). A volume of 1 mL of sodium citrate buffer was used to dissolve every 10 mg of STZ. The solution was mixed well by blowing on ice and used immediately.

② Modeling method and standard: A total of 24 male SD rats, 4–6 weeks old and of SPF grade, were obtained from the animal laboratory of Linyi Municipal People’s Hospital. After a 1-week acclimatization feeding period, the rats were switched to a high-fat chow diet for 4 weeks. Tail vein blood glucose and body weight were measured and recorded for each rat. Following a 10–12 h fasting period with no water access, each rat received a single intraperitoneal injection of a pre-prepared STZ solution at a dose of 50 mg/kg. Tail vein blood glucose was measured again 6 days after injection. Rats with two fasting blood glucose readings ≥11.1 mmol/L or a single random blood glucose reading ≥16.7 mmol/L were considered diabetic models. Concurrently, diabetic rats were observed for symptoms of diabetes, including changes in behavior, increased thirst, increased urination, and changes in stool consistency. Throughout the experimental period, the rats were maintained on a high-fat diet.

③ Rats were anesthetized with an intraperitoneal injection of 2% pentobarbital sodium solution at a dose of 0.3 mL/100 g body weight. Following anesthesia, the skin on the back of the rats was prepared. The surgical area was disinfected routinely. A 12-mm diameter perforator was used to create a full-thickness skin defect on each side of the spine at the same level on both sides of the back of each rat. The distance between the two defects was sufficient to allow for complete skin removal until the underlying muscle was exposed. To prevent postoperative self-mutilation, the rats were housed individually.

④ In the experimental group, cultured P3 generation ADSCs were injected at multiple points around each wound and base tissue. Approximately 5 × 105 ADSCs were injected per wound. In the control group, an equal volume of PBS buffer was injected. Following injection completion, a sterile cotton ball was gently pressed at the injection site to promote uniform distribution of the injected material. On days 0, 3, 7, 10, and 14 following wound creation, photographs of the dorsal wounds of the rats were taken at a perpendicular angle. The images were analyzed using ImageJ software (NIH, Bethesda, MD) to determine the wound healing rate, which was then statistically analyzed. Wound healing rate (WHR) was calculated as: WHR = (initial measurement of wound area - day N wound area)/initial measurement of wound area × 100%.

⑤ Wound sampling: Rats from the 7-day group were euthanized, and the surgical area was disinfected. Wound tissue samples were obtained from the center of the wound and the surrounding 2 mm of full-thickness skin. The tissue was then divided into two equal parts. One part was placed in 4% paraformaldehyde for subsequent HE and Masson staining after 24 h. The remaining tissue samples were placed in a freezing tube and stored at −80°C. In this experiment, the samples of the ADSC group and the control group were marked with serial numbers, and the samples were selected by random selection for the experiment. Four samples were selected from the ADSC group and the control group for 4D-DIA quantitative proteomics analysis.

### 2.5 4D-DIA quantitative proteomic analysis and data processing

In a frozen state, samples were thawed on ice and then suspended in protein lysis buffer (8M urea, 1% SDS) supplemented with appropriate protease inhibitors to prevent protein degradation. The mixture was then homogenized using a high-flux tissue grinding machine for three cycles of 40 s each. Following homogenization, the mixture was incubated on ice for 30 min with brief vortexing (5–10 s) every 5 min. The homogenate was then centrifuged at 16,000 × g for 30 min at 4°C. Protein concentration in the collected supernatant was subsequently determined using the Bicinchoninic Acid (BCA) method with a commercially available BCA Protein Assay Kit (Thermo Scientific) according to the manufacturer’s instructions. Finally, protein samples were quantified and subjected to SDS-PAGE electrophoresis.

100 μg of protein were resuspended in 100 mM Triethylammonium bicarbonate buffer (TEAB). The mixture underwent reduction with 10 mM Tris(2-carboxyethyl)phosphine (TCEP) for 60 min at 37°C, followed by alkylation with 40 mM iodoacetamide (IAM) for 40 min at room temperature in darkness. The solution was then centrifuged at 10,000 × g for 20 min at 4°C. The resulting pellet was resuspended in 100 mM TEAB, and trypsin was added at a 1:50 trypsin-to-protein mass ratio. The mixture was incubated for digestion at 37°C overnight. After digestion, peptides were separated from the solution by vacuum pump. The enzymatically digested peptides were then resuspended in 0.1% trifluoroacetic acid (TFA) and desalted with HLB cartridges before being dried using a vacuum concentrator. Finally, the purified peptides were quantified using the Thermo Fisher Scientific Peptide Quantification Kit (item #23275).

Following quantification, the peptides were redissolved in a liquid chromatography-mass spectrometry (LC-MS) loading buffer composed of 2% acetonitrile (ACN) with 0.1% formic acid. This buffer also contained appropriate internal reference time (iRT) peptides for retention time calibration. The analysis was performed using an EASY-nLC system (Thermo, United States) coupled with a timsTOF Pro2 mass spectrometer (Bruker, Germany) at Majorbio Bio-Pharm Technology Co. Ltd (Shanghai, China). Briefly, a C18-reversed phase column (75 μm × 25 cm, Ionopticks, United States) was equilibrated with two solvents: solvent A (2% ACN with 0.1% formic acid) and solvent B (80% ACN with 0.1% formic acid). The elution of peptides employed a gradient: 0–45 min, 3%–28% B; 45–50 min, 28%–44% B; 50–55 min, 44%–90% B; 55–60 min, hold at 90% B. The tryptic peptides were separated at a flow rate of 250 nL/min.

Data-independent acquisition (DIA) data were acquired using a timsTOF Pro2 mass spectrometer operated in DIA-PASEF mode. MS data were collected over an m/z range of 400–1200 and an ion mobility range of 0.57–1.47 Vs.·cm⁻^2^. Both accumulation time and ramp time were set to 100 m. During MS/MS data collection, each tims cycle contained one MS and ten PASEF MS/MS scan. Exclusion was active after 0.4 min. A total of 64 DIA-PASEF windows were used (25 Th isolation windows).

Spectronaut software (Version 14) was used to search the DIA-PASEF raw data. Retention times were corrected by iRT and six peptides per protein and 3 daughter ions per peptide were selected for quantitative analysis. The parameters are as follows up: Protein FDR≤0.01, Peptide FDR≤0.01, Peptide Confidence ≥99%, XIC width≤75ppm. The shared peptides and modified peptides were excluded, and the peak areas were calculated and summed to give the quantitative results. Only the proteins which has at least one unique peptides were used for protein identifications.

Bioinformatic analysis of proteomic data was performed with the Majorbio Cloud platform (https://cloud.majorbio.com). *P*-values and fold changes (FC) for the proteins between the two groups were calculated using R package “*t*-test”. A threshold of absolute fold change greater than 1.2 or less than 0.83, along with a *P*-value less than 0.05, was applied to identify differentially expressed proteins (DEPs). Functional annotation for all identified proteins was conducted using the Gene Ontology (GO) database (http://geneontology.org/) and the Kyoto Encyclopedia of Genes and Genomes (KEGG) pathway database (http://www.genome.jp/kegg/). DEPs were then used for further enrichment analysis within both GO and KEGG pathways. PPI analysis was performed using the STRING database (version 11.5).

### 2.6 ROC curve analysis and Western blot (WB) experiments to verify the target proteins

#### 2.6.1 Statistical analysis

Data were statistically analyzed using SPSS 26.0 software. Measurements were expressed as mean ± standard deviation (SD), and comparisons between the ADSC group and the control group were made using the independent samples *t*-test, with a *p*-value of less than 0.05 considered statistically significant.

## 3 Results

### 3.1 Morphology and characterization of ADSCs

#### 3.1.1 Morphology of ADSCs

**FIGURE 1:**
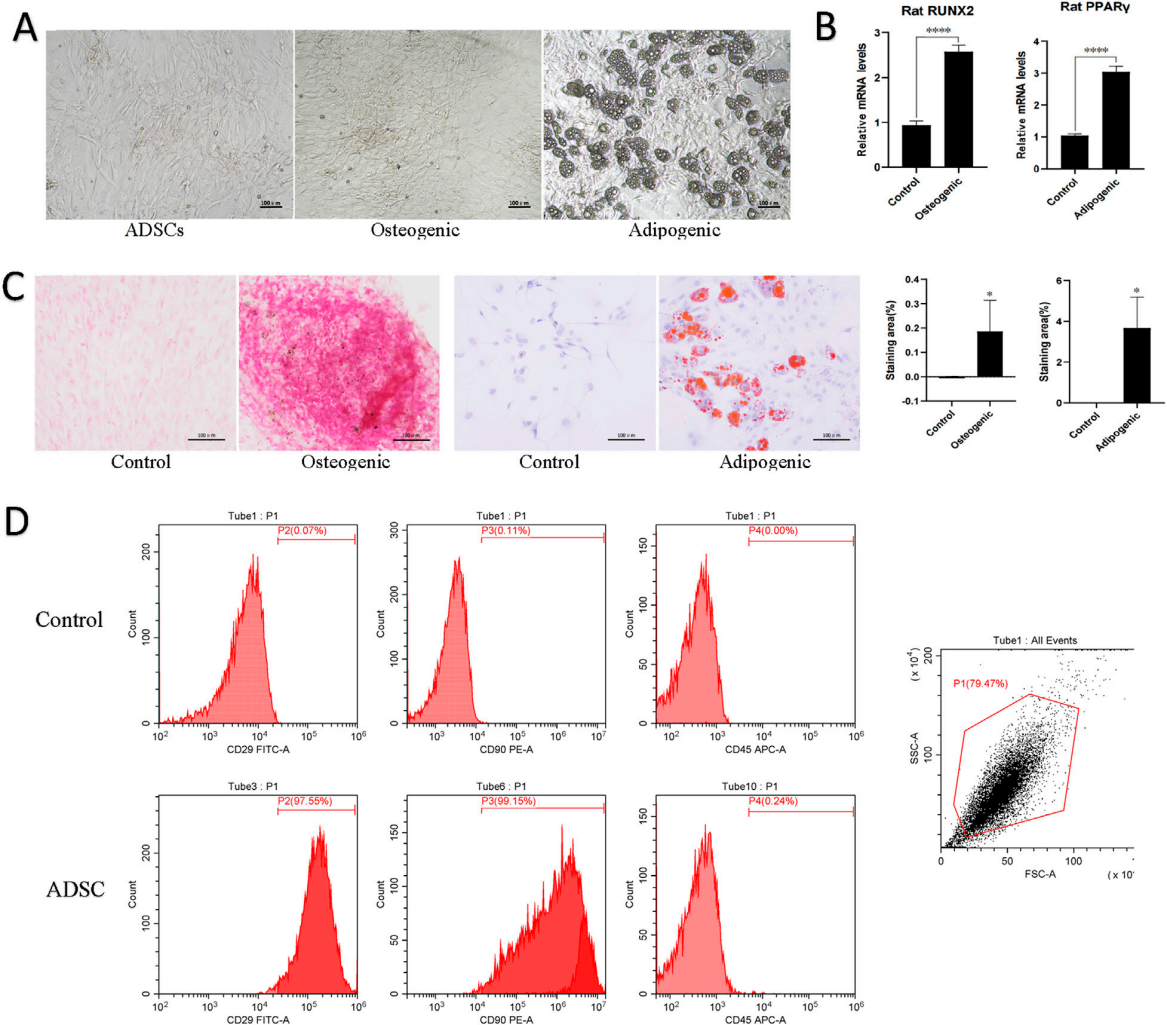
Morphology and characterization of ADSCs. **(A)** Cell morphology of normal ADSCs, osteogenesis and adipogenic induction of ADSCs under inverted microscopy. Scale bars, 100 μm. **(B)** Quantitative PCR results for lineage-specific markers (PPARγ for adipogenesis and RUNX2 for osteogenesis). ****p < 0.001. **(C)** Following the induction of ADSCs lipogenesis, a red oil O staining procedure was performed, which revealed the presence of red lipid droplets. Similarly, a red or orange-red precipitate was observed following the induction of ADSCs osteogenesis, as evidenced by alizarin red staining. **(D)** Flow cytometry was employed to identify the presence of CD molecules on the surface of ADSC, which exhibited positive expression of CD29 and CD90 and negative expression of CD45.

At 24 h post-inoculation, cells in primary culture exhibited partial adherence to the wall, followed by a gradual elongation process that resulted in the formation of spindle-shaped cells. After changing the culture medium, the adherent cells were predominantly ADSCs displaying long, shuttle-forming fibroblast characteristics. By the third generation, the ADSCs exhibited a tightly arranged, radial morphology under an inverted microscope, consistent with fibroblast-like cells (Figure 1A).

#### 3.1.2 Identification of ADSCs multidirectional differentiation capacity and flow cytometry results

Following the induction of lipogenesis in ADSCs, the cells underwent a morphological change, transitioning from a long spindle shape to a shorter, more contracted morphology. This change was accompanied by the appearance of intracellular lipid droplets, which stained red with oil red O staining, indicative of substantial lipid deposition within the cells. In contrast, upon osteogenic induction, ADSCs exhibited a distinct morphological shift. Initially elongated and spindle-shaped, the cells gradually transformed into a polygonal morphology. Additionally, cell aggregation and a surface layer of secretion were observed. Furthermore, intracellular calcium salt deposition and the presence of red or orange-red intracellular calcium nodules were evident after alizarin red staining, signifying the positive osteogenic differentiation of the cells. In addition, quantitative PCR of lineage-specific markers (PPARγ for adipogenesis and RUNX2 for osteogenesis) showed significant differences between the experimental and control groups (*p* < 0.001). These observations collectively demonstrate the capacity of the P3 generation ADSCs to differentiate into at least two distinct mesodermal lineages (Figures 1B,C).

Flow cytometry analysis of the P3 generation ADSC demonstrated characteristic markers of MSCs. Specifically, CD29 and CD90 exhibited strong positivity (exceeding 97%), while CD45 expression was minimal (less than 0.25%). These findings were consistent with the identification of the cultured cells as MSCs (Figure 1D).

### 3.2 Mold formation and dorsal wound healing in diabetic rats

Following acclimation, twenty-four rats were successfully induced with diabetes using STZ injection. Prior to drug administration, no significant abnormalities were observed in the general health of the rats. However, consistent with established diabetic symptoms, the rats developed polydipsia, polyphagia, polyuria, and loose stools after modeling. Additionally, decreased mobility was noted. Throughout the modeling process, blood glucose levels were consistently maintained at or above 16.7 mmol/L. Sixteen rats were then randomly assigned to two groups for the subsequent dorsal wound healing experiment: the 7-day group (n = 8) and the 14-day group (n = 8).

#### 3.2.1 Wound healing results

**FIGURE 2:**
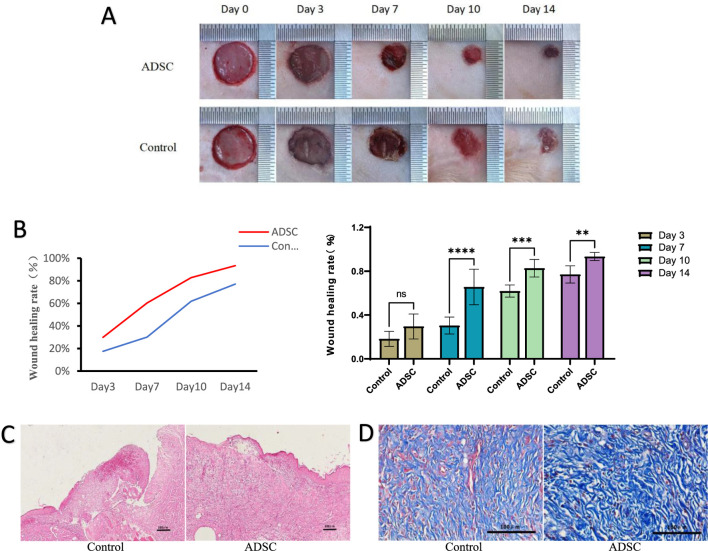
Wound healing **(A)** Observation of wound healing in each group. **(B)** Statistical analysis of healing rate at each time point in each group, line graph of healing rate (left), bar graph of healing rate (right), ns *p* > 0.05, *p < 0.05, **p < 0.01, ***p = 0.001, ****p < 0.001. **(C)** The results of HE staining (×100 magnification). **(D)** The results of Masson staining (×400 magnification).

In all measured days postoperatively, the wound healing rate was significantly higher in the ADSC group than in the PBS group (day 7: *p* < 0.001, day 10: *p* = 0.001, day 14: *p* < 0.01), except on day 3 (*p* > 0.05) (Figures 2A,B). Specifically, on postoperative days 3, 7, 10, and 14, the healing rates in the ADSC group were 29.90% ± 10.41%, 65.07% ± 12.38%, 82.76% ± 8.04%, and 93.39% ± 3.74%, respectively, compared to 17.51% ± 6.91%, 30.09% ± 11.22%, 61.85% ± 5.54%, and 77.07% ± 7.83% in the PBS group.

#### 3.2.2 Histological observations

(1) HE staining on postoperative day 7 revealed superior wound healing in the ADSC group compared to the control group (Figure 2C). The ADSC group exhibited enhanced epidermalization at the wound margins, suggesting faster re-epithelialization. Additionally, the margins showed signs of early skin appendage formation, indicating a more complete regeneration process. Furthermore, the ADSC group displayed a more robust skin structure and a greater density of newly formed capillaries within the granulation tissue, potentially facilitating tissue repair. In contrast, the center of the trauma in the control group remained primarily composed of dermal tissue, suggesting a slower healing process.
(2) Masson staining: Microscopic examination revealed a generally greater deposition of collagen in the ADSC group compared to the control group throughout the observation period. This difference was most pronounced on postoperative day 7, where the ADSC group exhibited clustered collagen deposition in the dermis, exceeding that of the control group to a statistically significant degree (Figure 2D).

### 3.3 4D-DIA quantitative proteomic analysis results

#### 3.3.1 Identification of differentially expressed proteins

Extracted from peritraumatic granulation tissue as detailed in the “Materials and Methods” section, protein samples were determined to be of sufficient quantity based on quantification (Table 1) and exhibited clear, undegraded bands on SDS-PAGE (P.S. 1). These observations indicated that the protein sample was acceptable, but only met the needs of one subsequent 4D DIA experiment, and therefore the protein sample met the standard of grade B.

**TABLE 1:** Protein concentrations in the samples.

Serial No.	Sample name	Protein concentration (㎍/μl)	Total protein (㎍)	Quality control results
1	ADSC1	6.302	2710	B
2	ADSC2	4.508	1938	B
3	ADSC3	5.803	2495	B
4	ADSC4	5.899	2537	B
5	PBS1	7.309	3143	B
6	PBS2	7.157	3078	B
7	PBS3	4.808	2067	B
8	PBS4	4.092	1760	B

#### 3.3.2 Screening of DEPs

**FIGURE 3:**
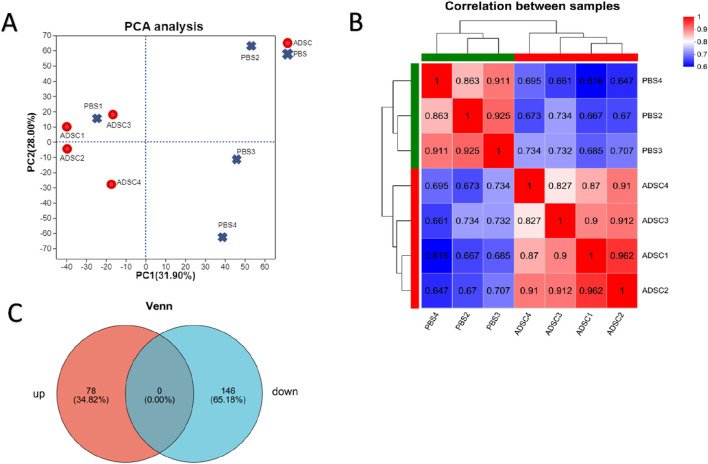
4D-DIA quantitative proteomic analysis reveals alterations in protein expression following ADSC intervention in diabetic rats with dorsal trauma. **(A)** Principal Component Analysis (PCA) scores are used to represent the distance between each sample point, with closer distances indicating a higher degree of similarity between the samples. **(B)** The sample correlation heat map illustrates the correlation between two samples, with each grid representing the correlation coefficient between the samples. The color depth of the grid represents the magnitude of the correlation coefficient, with red indicating a strong correlation and blue indicating a weak correlation. This analysis confirms that the bias between the samples is minimal, indicating that the experimental results are reliable. **(C)** Venn diagrams illustrate the number of unique and shared proteins between the ADSC and control groups, with the overlap representing the number of proteins shared between the two groups.

**FIGURE 4:**
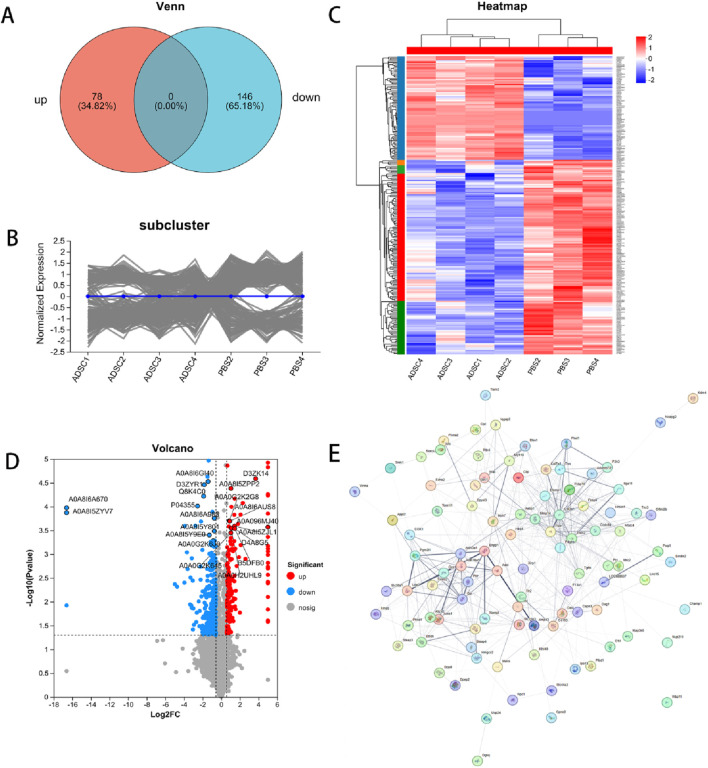
Differentially expressed proteins. DEPs in ADSC and control groups were shown by expression Venn diagram **(A)**, pattern clustering analysis (including heatmap and subcluster trend plot) **(B, C)** and volcano plots **(D)** after PPI screening **(E)** based on log2 multiplicity of difference (FC ≥ 1.5 or FC ≤ 0.67) and *P*-value < 0.05 as screening criteria.

According to the scatter plots of principal component analysis (PCA) (Figure 3A), the PC1 and PC2 values of PBS1 were mixed with the ADSC group and could not be separated from the samples of the ADSC group, so PBS1 was excluded. After the exclusion of the abnormal sample PBS1, there was a clear separation between the ADSC group and the control sample according to the sample correlation heatmaps (Figure 3B). This analysis identified a total of 4803 proteins in the experiment (Figure 3C). Further quantitative analysis of the ADSC and control groups revealed 474 DEPs. Through PPI network construction and analysis, 224 key proteins were identified: 78 exhibiting increased expression and 146 exhibiting decreased expression in the ADSC group compared to the controls (Figure 4; Table 2).

**TABLE 2:** List of differential proteins.

Accession	Symbol	Log2FC	*P*_value	Regulate
D3ZK14	Tnn	3.693	<0.001	up
A0A8I5ZPP2	Thy1	1.043	<0.001	up
A0A0G2K2G8	Tpm4	0.8568	<0.001	up
A0A8I6AUS8	Dpysl3	0.8992	<0.001	up
A0A8I5ZJL1	Myh10	1.421	<0.001	up
A0A096MJ40	Hmcn1	5	<0.001	up
D4A8G5	Tgfbi	1.354	<0.001	up
A0A0H2UHL9	Dbn1	1.075	<0.001	up
B5DFB0	P3h3	1.439	<0.001	up
A0A0G2K645	Wls	1.035	<0.001	up
A0A0G2JZ30	Fam76a	5	<0.001	up
A0A8I6A0T8	Serpinb9	0.6462	<0.001	up
A0A8I6A876	Wdr36	5	<0.001	up
Q5U2V1	Fkbp10	1.285	<0.001	up
P35053	Gpc1	1.124	<0.001	up
A0A8I6APN3	Txndc5	0.7924	<0.01	up
D4A2G6	Thbs2	1.895	<0.01	up
P24368	Ppib	0.6406	<0.01	up
Q8R5M3	Lrrc15	1.655	<0.01	up
Q9JKL7	Srek1	5	<0.01	up
A0A8I6GI40	Ubac1	−1.42	<0.001	down
D3ZYR1	Fcho2	−1.835	<0.001	down
Q8K4C0	Fmo5	−1.893	<0.001	down
P04355	Mt2	−2.55	<0.001	down
A0A8I6A670	Ipo13	−16.61	<0.001	down
A0A8I5ZYV7	Mrpl21	−16.61	<0.001	down
A0A8I6A983	Pdcd2	−0.7444	<0.001	down
A0A8I5Y801	Etfdh	−0.7454	<0.001	down
A0A8I5Y9E0	Tspan8	−1.289	<0.001	down
A0A0G2K6J0	Steap4	−0.8765	<0.001	down
A0A0G2K4E2	Krt34	−1.048	<0.001	down
A0A0G2K0I3	Nampt	−0.7058	<0.001	down
Q7TNZ9	Cmtm6	−0.816	<0.001	down
Q499V1	Upp1	−0.616	<0.001	down
D3ZSA0	Anxa9	−0.7939	<0.001	down
A0A8I6AF07	Cab39l	−1.05	<0.001	down
M0RAD5	Clpp	−0.8751	<0.01	down
A0A0H2UI07	Pklr	−1.001	<0.01	down
A0A8I6AL97	Npc1	−0.7326	<0.01	down
F1LXD8	Emilin2	−1.241	<0.01	down

Note: The top 20 DEPs, for each of up- and downregulation are labeled in order of significance.

#### 3.3.3 Protein function annotation enrichment analysis results

**FIGURE 5:**
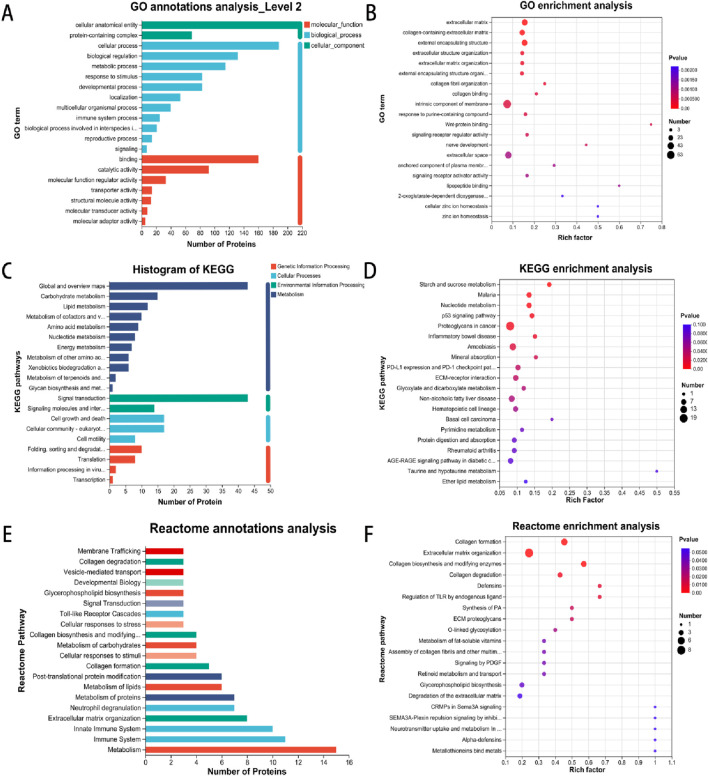
Enrichment analysis of GO, KEGG and Reactome pathways involved in DEP. **(A)** GO annotations indicate the multilevel classification of the selected protein set, with the horizontal coordinate representing the name of the classification and the vertical coordinate representing the number of proteins in the set that have been annotated to that classification. **(B)** GO enrichment analysis employs a horizontal coordinate to represent the enrichment rate, with a larger ratio indicating a greater degree of enrichment. The vertical coordinate represents the GO term, with the parameter being the *P*-value. **(C)** KEGG annotations present the name of the metabolic pathway in the horizontal coordinate and the number of proteins annotated to the pathway in the vertical coordinate. **(D)** The horizontal coordinate in KEGG enrichment analysis represents the enrichment rate, with a larger ratio indicating a greater degree of enrichment. The vertical coordinate is the KEGG pathway, with the parameter being the *P*-value. **(E)** The vertical coordinate represents the name of the Reactome metabolic pathway, while the horizontal coordinate denotes the number of proteins that have been annotated to the pathway. **(F)** Reactome enrichment analysis is depicted on a vertical axis, where the Reactome pathway name is indicated. On a horizontal axis, the enrichment rate is displayed, with a larger rich factor indicating a greater degree of enrichment.

To characterize the subcellular localization, molecular function, and biological processes associated with DEPs following ADSC intervention in diabetic rat dorsal wounds, three GO categories were assessed: Cellular Component (CC), Molecular Function (MF), and Biological Process (BP). GO analysis of the annotated DEPs revealed significant enrichment of terms related to “cellular anatomical entity” and “protein-containing complex” within the cellular component category (Figure 5A). Similarly, the most significant enrichments in the molecular function category were associated with “binding” and “catalytic activity”. In the biological process category, the most enriched terms were “cellular process” and “biological regulation”. Further analysis identified the top 20 most enriched GO terms. Within the molecular function category, “Wnt-protein binding” was the most enriched term, with upregulated proteins such as Sfrp2, Cthrc1, and Wls demonstrating this function. Conversely, terms like “lipopeptide binding” (downregulated proteins Tlr2, Cd14, Cd1d1) and “nerve development” (downregulated proteins Ctsl, Dag1, Afg3l2) were also significantly enriched in the molecular function and biological process categories, respectively. Interestingly, the cellular component analysis revealed significant enrichment for terms related to the extracellular matrix, including “extracellular matrix”, “collagen-containing extracellular matrix”, and “external encapsulating structure” (Figure 5B).

KEGG pathway analysis of the DEPs identified enrichment in pathways associated with cellular signaling, including starch and sucrose metabolism, nucleotide metabolism, the p53 signaling pathway, and extracellular matrix (ECM) receptor interaction (Figures 5C, D).

Consistently, Reactome pathway analysis revealed a predominance of DEPs associated with metabolic and immune system pathways. Further enrichment analysis using Reactome identified a focus on signaling pathways involved in collagen formation, extracellular matrix organization, and collagen biosynthesis and modifying enzymes (Figures 5E, F).

#### 3.3.4 Screening and Western blot assay to validate target proteins

**FIGURE 6:**
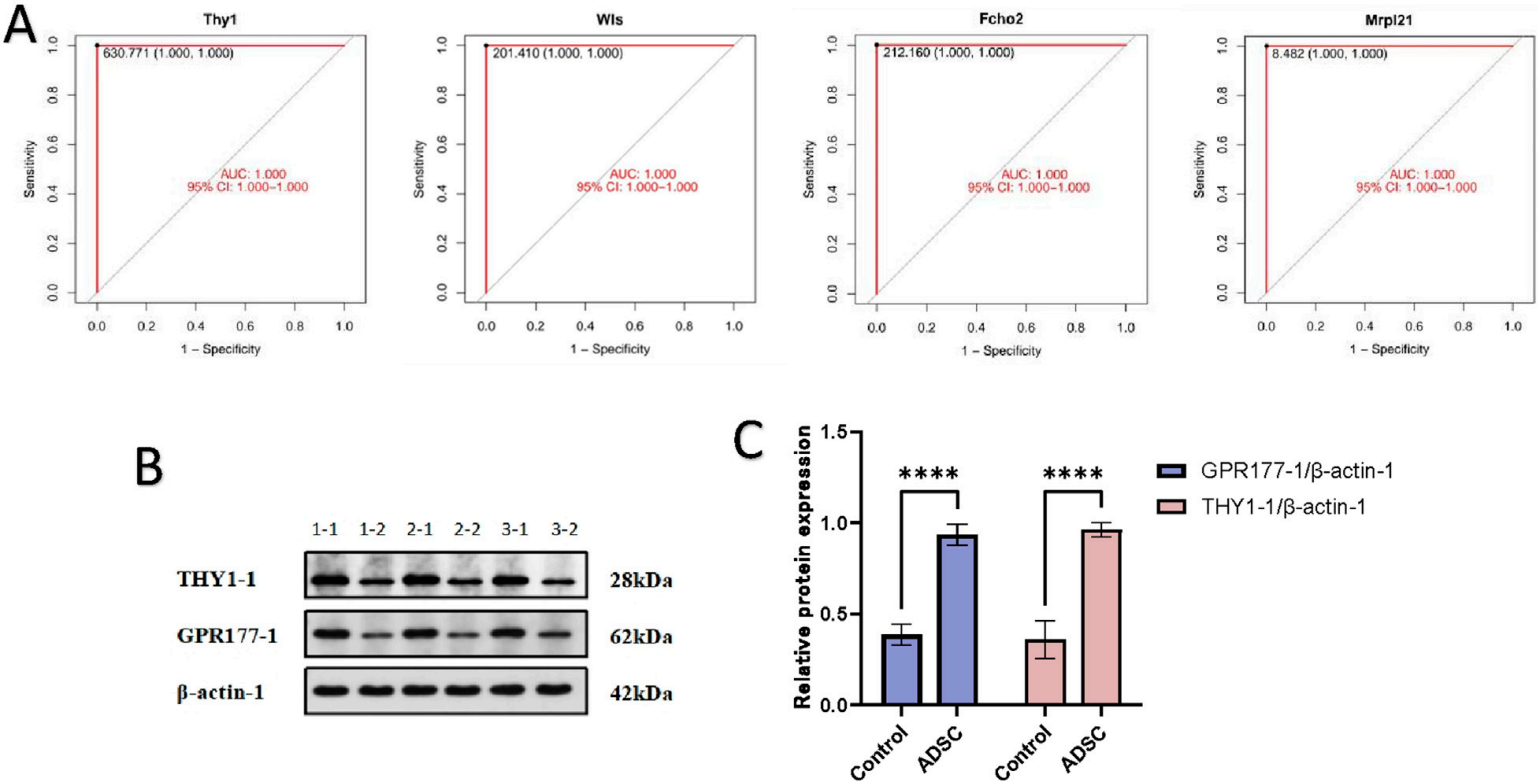
Verification of target protein **(A)** The results of the ROC analysis are presented in the form of a graph, with the horizontal coordinate representing the false-positive rate (1-Specificity %) and the vertical coordinate representing the sensitivity (Sensitivity %), which is equivalent to the true-positive rate. The area under the curve (AUC) is also displayed, along with the 95% confidence interval (95% CI). **(B)** WB electrophoresis strip chart. 1-1, 2-1, 3-1 were the ADSC group; 1-2, 2-2, 3-2 were the control group. **(C)** Expression of THY1-1 and GPR177-1 in each group: The expression of THY1 and GPR177-1 proteins in the ADSC group was demonstrably greater than that in the control group, with a statistically significant difference (****p < 0.001).

Following analysis of the PPI network using the LASSO plugin, four crucial node genes were identified. Among these, THY1 (CD90) and WLS (GPR177-1) were upregulated in the ADSC group compared to the control, while Fcho2 and Mrpl21 were downregulated. The diagnostic potential of these four proteins was assessed using receiver operating

characteristic (ROC) curve analysis. The resulting area under the curve (AUC) was 1.000 with a 95% confidence interval of 1.000–1.000, indicating excellent discriminatory power (Figure 6A).

To validate these findings, peritracheal tissues were harvested on day 7 and subjected to Western blot analysis. Consistent with the *in silico* analysis, Western blot experiments demonstrated significantly higher expression levels of THY1 and WLS in the ADSC group compared to the PBS group (*p*-value <0.001) (Figures 6B, C).

## 4 Discussion

MSCs have established themselves as a cornerstone of injury repair and tissue engineering (Wang et al., 2015). In recent years, ADSCs have emerged as a particularly exciting research area due to their readily available and abundant nature.

Compared to MSCs, ADSCs offer significant advantages in both scientific research and clinical applications. Firstly, ADSCs are easier to isolate and more plentiful, potentially making them a superior source of stem cells for wound repair and regeneration (Zuk et al., 2002). Secondly, studies have demonstrated that ADSCs contribute to the wound healing process by secreting growth factors and cytokines that promote skin repair and regeneration, while also possessing the ability to differentiate into other cell lineages and types (Mazini et al., 2020). Consequently, the utilization of proteomics and genomics combined with bioinformatics for functional analysis and specific marker identification following ADSC intervention in various diseases and trauma-induced wounds represents a major focus of basic scientific research.

Proteomics, a field that integrates laboratory techniques with bioinformatics, has gained significant traction in recent years due to its broad applicability in basic scientific research and clinical trials. This powerful approach facilitates the identification of a multitude of disease-related protein markers and the generation of protein profiles specific to detected diseases. Consequently, proteomics plays a vital role in uncovering biological markers for various pathologies. Prior studies have successfully employed proteomic analysis of MSCs to elucidate key proteins and critical pathway activation during damage repair and induced differentiation, laying a solid foundation for the clinical application of stem cell therapy (Mazini et al., 2020; Elia et al., 2020; Zvonic et al., 2007). Notably, past research in this domain has primarily focused on bone marrow-derived MSCs and ADSCs, with the research content centered on the repair of soft tissues, organs, and other post-injury conditions (Ouyang et al., 2018; Yu et al., 2016). Building upon this knowledge base, the present study aims to utilize similar detection methods to screen for differential markers and analyze their associated biological functions and signaling pathways following ADSC intervention in diabetic rat wounds. This investigation seeks to establish a theoretical foundation for subsequent clinical trials and therapeutic interventions. Ultimately, the successful outcome of this study has the potential to guide effective preventative measures for diabetic wounds, accelerate ulcer healing, and reduce amputation rates in diabetic patients (particularly toe amputations).

In this study, 4D-DIA quantitative proteomic technology was employed to analyze the untagged quantitative protein profiles of peritracheal tissues from experimental and control groups. This analysis resulted in the initial identification of 474 differentially expressed proteins. Following further screening, 224 differentially expressed proteins were identified, with 78 being upregulated and 146 downregulated. Examples of upregulated proteins included Tnn, Thy1, Tpm4, Dpysl3, Myh10, Hmcn1, Tgfbi, Dbn1, P3h3, and Wls, while examples of downregulated proteins included Ubac1, Fcho2, Fmo5, Mt2, Ipo13, Mrpl21, Pdcd2, Etfdh, Tspan8, and Steap4.

GO analysis revealed that the primary molecular function associated with the identified DEPs is related to Wnt protein binding. Wnt proteins are secreted glycoproteins that play a critical role in regulating cell growth, development, and differentiation. They function in target cells by activating various Wnt signaling pathways through autocrine or paracrine signaling, which is initiated by binding to corresponding receptors. There are four major Wnt signaling pathways: the classical Wnt/β-catenin pathway, the non-classical Wnt pathway, the Wnt/Ca2+ pathway, and the Wnt/PCP pathway (Lorenowicz and Korswagen, 2009). The Wnt/β-catenin pathway is the most extensively studied among these four. The Wnt/β-catenin signaling pathway is a vital signaling cascade in the human body, intimately linked to embryonic development. It plays a crucial role in regulating various biological processes, including cell growth, proliferation, differentiation, and apoptosis. Under normal physiological conditions, the Wnt signaling pathway governs many essential aspects of cell growth, development, and differentiation. It also contributes significantly to embryonic development and tissue formation and function in adults. However, disruptions or abnormalities within this pathway can lead to the development of various diseases.

Thy1 (CD90), a glycosylated and glycophosphatidylinositol (GPI)-anchored membrane protein, has emerged as a distinct marker for various stem cell populations. Notably, Thy1 expression is enriched in epidermal basal cells (Koren et al., 2022) and has been identified in multiple tissues as a marker of stem cells (An et al., 2018; Gargett, 2006; Yovchev et al., 2008). This protein is highly expressed in MSCs and specific fibroblast subsets, where it plays a critical role in maintaining epidermal homeostasis and repair. Mechanistically, Thy1 has been shown to inhibit epidermal YAP activity through a convergent molecular pathway. Deletion of Thy1 leads to the dissociation of the adherens junction complex, consequently triggering YAP release and translocation into the nucleus. Interestingly, Thy1-deficient mice exhibit enhanced wound repair and hair follicle regeneration, a phenomenon attributed to increased YAP-dependent proliferation. In conclusion, Thy1 acts as a key regulator of cell-matrix and cell-cell interactions, controlling YAP activity and thereby influencing skin homeostasis and regeneration. Conversely, epidermal YAP activity appears to drive classical Wnt16/β-catenin signaling, promoting the proliferation of keratinizing cells in both *in vitro* and *in vivo* models (Sedov et al., 2022; Mendoza-Reinoso and Beverdam, 2018).

This study also revealed that Wls, a protein exhibiting significant differential expression, is essential for the secretion of various Wnt ligands. Notably, the absence of Wls in epithelial cells disrupts the activation of Wnt/β-catenin signaling in neighboring epidermal melanocytes. These findings highlight the crucial role of exogenous Wnt ligands in initiating Wnt/β-catenin signaling within melanocytes of hair follicle derivatives during the wound healing process. Wls, a highly conserved seven-pass transmembrane protein, interacts with Wnt, and its presence is critical for Wnt secretion regardless of the specific signaling pathway involved. In the absence of Wls, Wnt becomes retained within its producing cells, rendering it nonfunctional. Interestingly, all Wnt signaling pathways appear to require the involvement of MOM-3 (also known as MIG-14), the direct homolog of Wls (Rie et al., 2021). Supporting this notion, studies by Hausmann et al. (Hausmann et al., 2007) and Michaux et al. (MichauxMichaux and Le Borgne, 2009) have demonstrated that Wls is specifically required for Wnt protein secretion, with no significant effect on the secretion of other proteins observed in *ex vivo* Wls gene disruption experiments using Hu cells. Concurrently, the discovery of Wls’s role coincided with the identification of the retromer complex as another crucial component for Wnt protein secretion. Wnt proteins, Wls, and the retromer complex function together in a dynamic cyclical manner: Wls facilitates Wnt secretion, while the retromer complex sorts and recycles Wls in a state of equilibrium. This coordinated activity ensures the proper secretion of Wnt proteins (Franch-Marro et al., 2008). Disrupting the balance within this cycle can potentially interfere with the functional secretion of Wnt proteins and consequently modulate Wnt pathway activity in target cells.

This study also revealed significant enrichment in the subcellular localization category for terms related to the external envelope structure, ECM, and collagen-containing extracellular matrix. As advancements in cell separation technology and molecular biology progress, the understanding of the ECM has evolved beyond its role as a mere cellular scaffold. It is now recognized that the ECM provides crucial biochemical and biophysical cues, significantly impacting cell morphology, growth, division, differentiation, and apoptosis (Toole, 2004). Furthermore, the ECM has been demonstrably linked to various physiological processes, including immunity, inflammation, wound healing, angiogenesis, and malignant transformation. DFU wound healing encompasses cell migration, proliferation, and differentiation, alongside biological processes like ECM deposition and remodeling. The ECM, functioning as both structural support and a critical mediator of cellular interactions, plays a pivotal role in facilitating wound healing. Collagen, a major ECM component and key structural element of skin, can promote myofibroblast differentiation and fibrosis, thereby maintaining the natural ECM architecture and fostering healing (Seo et al., 2020). Therefore, a comprehensive investigation into the role and mechanisms underlying ECM homeostatic remodeling within the DFU microenvironment during wound healing has the potential to unveil novel research avenues for DFU therapeutic strategies targeting the ECM as a molecular target.

Wound healing is a complex and dynamic process that contributes to well-coordinated skin injury repair and involves many different types of cells, such as endothelial cells, fibroblasts, and keratinocytes (Bielefeld et al., 2013). In the context of diabetes, pathophysiological mechanisms involving cellular dysfunction, inflammation, hypoxia, neuropathy, impaired angiogenesis, and neovascularization impair the healing process (Guo and Dipietro, 2010)^.^ Recent studies have shown that ADSCs can improve wound healing by promoting fibroblast and keratinocyte migration and proliferation, collagen deposition, neovascularization, and macrophage polarization to the M2 phenotype (Hu et al., 2016; He et al., 2019; Ren et al., 2019; Shafei et al., 2020; Zhang et al., 2018). The pathways through which ADSCs promote diabetic wound healing are (Pomatto et al., 2021): the EGFR receptor (ERBB 2) signaling pathway and its downstream PI 3 K/Akt signaling cascade, which mediates various endothelial cell functions that are impaired during wound healing, including migration, proliferation, and survival leading to angiogenesis (Okonkwo et al., 2020); ECM-receptor interactions and adhesion junction pathways, which are essential for cell adhesion and motility; MAPK signaling pathways that regulate cell survival, differentiation, and proliferation; and the Wnt signaling pathway involved in cell proliferation and angiogenesis. Compared with protein analysis of ADSC-EV and BMSC-EV, ADSC-EV proteins showed higher interactions, with several pathways involved in angiogenesis (Wnt, FGF, EGF receptor, PDGF, TGFβ, and angiogenesis) (Pomatto et al., 2021), and ADSCs can promote cell proliferation, migration, and inhibit apoptosis in wound healing through Wnt/β-catenin signaling (Ma et al., 2019b). More mechanisms by which ADSCs promote wound healing are still to be explored.

The bioinformatic analysis of differentially expressed proteins following ADSC treatment of diabetic rat wounds in this experiment offers significant insights: firstly, it adopts a novel perspective by aligning with current scientific research trends. By analyzing the mechanism of diabetic wound healing by ADSCs at the protein level, this study provides a fresh approach to inform therapeutic research for this condition. Secondly, the identified key proteins Thy1 and Wls hold promise as potential novel targets for ADSC-based diabetic ulcer treatment. Finally, the experimental results suggest that ADSCs promote diabetic wound healing likely through activation of the Wnt/β-catenin signaling pathway, which regulates crucial biological processes like cell growth, proliferation, differentiation, and apoptosis. Furthermore, this pathway appears to contribute to ECM homeostasis remodeling, ultimately promoting diabetic wound healing.

## Data Availability

The raw data supporting the conclusions of this article will be made available by the authors, without undue reservation.
